# Origin of optical nonlinearity of photo-responsive liquid crystals revealed by transient grating imaging

**DOI:** 10.1038/s41598-019-42140-x

**Published:** 2019-04-08

**Authors:** Kenji Katayama, Daiki Kato, Kin-Ichiro Nagasaka, Minako Miyagawa, Woon Yong Sohn, Kuang-Wu Lee

**Affiliations:** 1 Department of Applied Chemistry, Chuo University, Tokyo, 112-8551 Japan; 2 PRESTO, Japan Science and Technology Agency (JST), Saitama, 332-0012 Japan; 3 Phase Transition Dynamics Group, Department of Physics, Kyoto University, Kyoto, Japan

## Abstract

A large optical nonlinearity has been observed for the photo-responsive liquid crystals under the condition that the nematic phase is close to the isotropic condition. The direct observation of the photo-response of a liquid crystal by the time-resolved transient grating phase imaging technique revealed that the optical nonlinearity was caused by the transiently generated phase formed inside the photo-induced isotropic region. A shock-like flow was observed for the formation of the transiently generated phase. Based on the theoretical calculation, we propose that a flow generated at the disordered/ordered interface induced the reorientation of the liquid crystal molecules, thereby generating a larger polarization and ultimately causing the optical nonlinearity.

## Introduction

Liquid crystals (LCs) have been studied for their unusually large response to optical stimulus^1–8^. There are many mechanisms responsible for their optical nonlinearities, ranging from ultrafast individual electronic photonic transition, to extremely slow molecular realignment in photosensitive (dye-doped) LCs, and they have been used in a large variety of photonic applications. The working principles underlying these NLO mechanisms and photonic applications have been the subject of many reviews and books^4–17^.

We have studied the dynamics of the photo-responsive LCs and LC polymers using the transient grating method on the nanosecond to second order^18,19^, where the refractive index change was monitored as a probe for the photo-response of the LCs. Disordering/reorientation or the phase transition/recovery processes were studied for N-(4-methoxybenzylidene)-4-butylaniline (MBBA) and dye-doped LCs^20^. We found that the time constants for the molecular rotation and the molecular ordering depended on the pump light intensity, and suggested that it was caused by the temporally formed domains^18^. In the reorientation process for azobenzene-doped 7-Heptyloxy-4-cyanobiphenyl (5CB), the reorientation time depended on the director axis, which was explained by the formation of the anisotropic domain^20^. Recently, we found that the molecular disordering during the phase transition propagates in a ballistic manner, with a speed of ~100 m/s^21^. In our previous work, the nonlinear refractive index change was observed when the temperature or the pump intensity were adjusted so that the phase was very close to the isotropic condition, and a drastic change in the dynamics was observed, which involved a long-lasting diffraction intensity on the order of 100 ms^22^.

In this report, we observed the photo-induced dynamics of an LC directly when the conditions for the huge optical nonlinearity was satisfied. For the direct observation of the dynamics, we have developed a phase imaging technique where a sample is irradiated by a stripe-patterned pump pulse. We found that a different phase, neither the nematic nor isotropic, was induced in the isotropic region, which is the origin of the optical nonlinearity.

## Theory and Method

**Figure 1:**
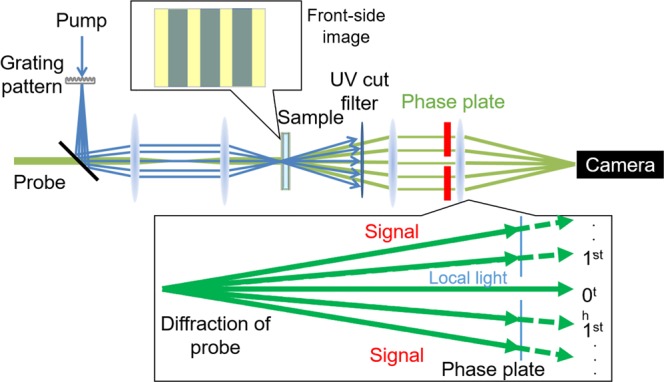
Optical setup for the transient grating (TG) imaging. A pump pulse was first passed through a grating pattern (fringe spacing = 75 μm), and the pattern of the image was formed at the sample position using a 4f configuration of two lenses with different focal lengths. The actual fringe on a sample was 36 μm. Another probe light was illuminated onto the same position of the sample, and the probe light is diffracted by a TG formed on the sample. For the imaging purpose, an additional optical setup was built to observe the phase image of the sample. To obtain the phase image, the 0th order light as a local light is mixed with the other diffraction order lights, where the phase of the other diffractions was controlled with a cover glass.

**Figure 2:**
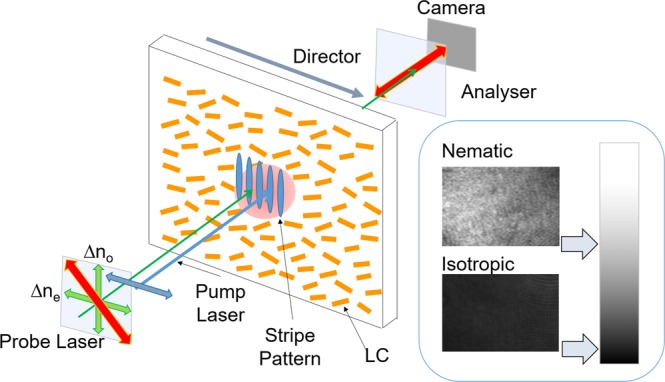
The sample configuration for the TG and TG imaging measurements. The pump fringe pattern was perpendicular to the director axis. The polarization of the pump light was set parallel to the director axis. When the probe polarization was parallel or perpendicular to the director axis, the TG signal intensity is proportional to (Δn_e_)^2^ or (Δn_o_)^2^. In the TG imaging, the probe light polarization was adjusted at 45 degrees from the ordinary/extraordinary direction and an analyzer was inserted in front of the camera to satisfy the cross-Nicole condition. Under this condition, the isotropic phase always gives dark image.

A phase imaging method was developed based on the transient grating (TG) method. The principle of the TG method was described in our previous paper^22^, and the additional difference is explained here. The optical configuration of the TG imaging is shown in Fig. 1. An excitation pulse passed through a grating pattern, and the pattern of the image was formed at the sample position using a 4f configuration of two lenses with different focal lengths and the actual fringe spacing on a sample was 1/2.1 of the original one. The pump pulse excites a sample, causing photo-isomerization and the subsequent molecular rotation, disordering, and release of heat. Since these processes induce a change in the refractive index, its pattern was the same as the pump-light pattern, called as transient grating (TG). In the TG measurement, the 1st-order diffraction intensity of the probe light is measured with a photodiode. As shown in Fig. 2, the director axis was set orthogonal to the stripe pattern. When the probe polarization was parallel to the director axis, the TG signal intensity is proportional to the square of the extraordinary refractive index, (Δn_e_)^2^. For the imaging purpose, additional 4f optical setup was built behind the sample to observe the phase image. A part of the diffraction light after passing through the sample was phase-shifted with a thin glass plate and was interfered with the rest of the light to make a phase image of the sample. In the TG imaging, a time sequence of phase images was obtained. The intensity at each pixel is proportional to the photo-induced phase change, which is proportional to a change in the refractive index.

When the probe light is diffracted by TG, the diffraction efficiency of the first diffraction light is equal to^23^:

1 $$\eta ={|{J}_{1}(\varphi )|}^{2}$$

where *J*_1_ is the first order Bessel function, and *ϕ* is the modulated phase in TG caused by the refractive index change, under the assumption for the thin grating condition. The modulated phase is:

2 $$\varphi =\frac{2\pi \Delta nd}{\lambda }$$

where d is the thickness of a sample, and $\lambda$ is the wavelength of a probe light. When the nonlinear optical effect is induced, the refractive index of a sample changes as:

3 $$n={n}_{0}+{n}_{2}I$$

where ${n}_{2}$ is the nonlinear refractive index, and $I$ is the pump light intensity, and ${n}_{2}I$ corresponds to the refractive index change in TG. From the dependence of the diffraction efficiency on the pump intensity, ${n}_{2}$ value is obtained.

In the case of LCs, the physical origin of the photo-induced refractive index change is typically divided into three components:

4 $${\rm{\Delta }}n(t)={\rm{\Delta }}{n}_{T}(t)+{\rm{\Delta }}{n}_{\rho }(t)+{\rm{\Delta }}{n}_{S}(t)$$

where the terms on the right side correspond to the index changes due to temperature, density, and the order parameter. In previous studies of the photo-induced dynamics of LCs, the ${\rm{\Delta }}{n}_{T}(t)$ term is smaller than ${\rm{\Delta }}{n}_{\rho }(t)$ and ${\rm{\Delta }}{n}_{S}(t)$ terms. The ${\rm{\Delta }}{n}_{\rho }(t)$ term is caused by the density change due to the disordering^9,11^, while the ${\rm{\Delta }}{n}_{S}(t)$ term is the ordering change of the LCs (i.e., the change in the order parameter). The last two components are the signal origins of the TG responses in the phase-transition process^18^. From Eqs (1) and (2), the intensity of the TG signal is proportional to the square of ${\rm{\Delta }}n({\rm{t}})$^24^.

In the TG imaging, the probe polarization direction was set 45 degrees from the ordinary/extraordinary direction, and an analyzer was inserted in front of the camera, which was oriented at 90 degrees to the probe polarization direction (cross-Nicole condition). For this configuration, the isotropic phase is always dark, and any other ordered state shows a brighter image than the isotropic image. Sample images for the nematic and the isotropic phases are shown in Fig. 2, where each phase was formed by control of the temperature (29 and 45 °C for the nematic and the isotropic phases, respectively) without the pump light.

For the simulation of the photo-induced change of LC, we performed a mesoscopic simulation based on the algorithm of liquid crystal stochastic rotational dynamics (LC-SRD)^25^. This approach uses a particle-based method and was first implemented by using the Ericksen-Leslie model for the evolution of LC bulk flows. In this study it is further generalized by using Q-tensor representation^26^. The model equations of Q-tensor nematic LC are:

5 $$\rho \frac{d{v}_{\beta }}{dt}={\partial }_{\alpha }(\,-\,p{\delta }_{\alpha \beta }+{\alpha }_{\alpha \beta }^{d}+{\alpha }_{\alpha \beta }^{f}+\alpha {\text{'}}_{\alpha \beta })$$

where ${\alpha }_{\alpha \beta }^{d}$, ${\alpha }_{\alpha \beta }^{f}$, and $\alpha {\text{'}}_{\alpha \beta }\,$are distortion, field-induced and viscous stress tensors. The detailed expressions can be found in the original paper^26^, and it is noted here the LC flows are directly coupled to pump laser via the field-induced stress ${\alpha }_{\alpha \beta }^{f}={\rho }_{e}\overrightarrow{{E}_{ext}}$. This field-induced stress is sometimes called Maxwell stress^27^, where the polarized charge ${\rho }_{e}=\nabla \cdot (\varepsilon \overrightarrow{{E}_{ext}})$ is a function of material dielectric property and external fields. The LC director, $\overrightarrow{d}$, advances in time by the torque generated by the strain tensor $(\nabla \overrightarrow{v}+{\nabla }^{T}\overrightarrow{v})/2$ and the vorticity tensor $(\nabla \overrightarrow{v}-{\nabla }^{T}\overrightarrow{v})/2$ under a shear flow. The direct optical torque, generated by the laser-induced field is considered as an extra term in the molecular field that aligns LC molecules. Based on single-elasticity assumption, the simplified governing equation for LC rotation by following:

6 $$\frac{\delta \overrightarrow{d}}{\delta t}=\frac{1}{2}(1-\frac{{\lambda }_{2}}{{\lambda }_{1}})\nabla \overrightarrow{v}\cdot \overrightarrow{d}-\frac{1}{2}(1+\frac{{\lambda }_{2}}{{\lambda }_{1}}){\nabla }^{T}\overrightarrow{v}\cdot \overrightarrow{d}-\frac{1}{{\lambda }_{1}}f(\overrightarrow{d})$$

where *λ*_1_ and *λ*_2_ are material parameters that determine whether LC is shear-tumbling or shear-aligning types. The last term $f(\overrightarrow{d})$ is the derivative of the Landau-de Gennes free energy, i.e. the molecular field that includes nematic alignment and optical torque by laser fields. All the LC material parameters are chosen for the MBBA type in this study^26^. The dielectric anisotropy is set to -0.11, and the Leslie viscosity coefficients were obtained from the experimental values at room temperature (298 K)^28^. The particle number density per cell was 70. This set of governing equations also reveals the Miesowicz viscosities due to LC configurations. It is worth noting that the field-induced stress tensor (Maxwell stress) is the source of electroconvection, and this is the driving mechanism of the laser-induced flows.

## Result and Discussions

**Figure 3:**
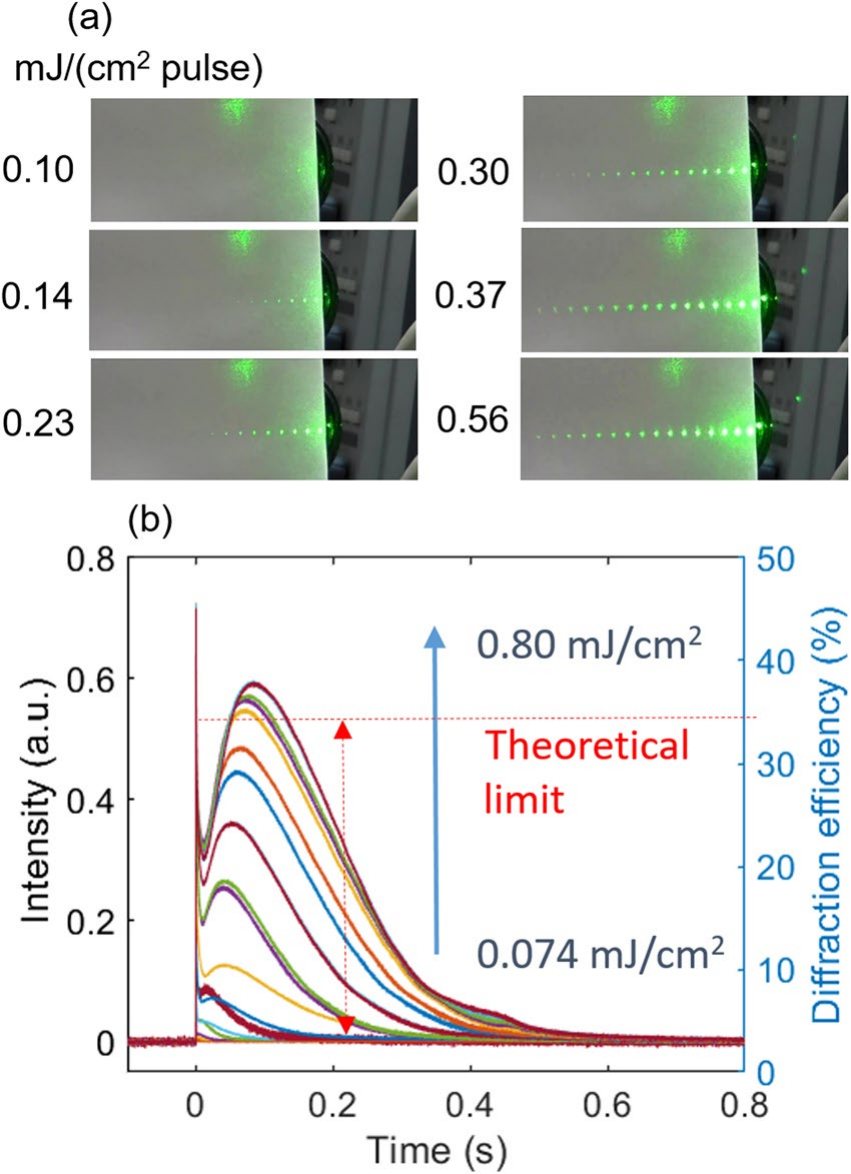
(**a**) Diffraction intensity patterns projected on a screen are shown for different pump intensities. (**b**) The transient grating (TG) responses for different pump intensities are shown for 0.074 to 0.80 mJ/cm^2^. The diffraction efficiency for the first order diffraction was shown on the right axis with the theoretical limit under the assumption of a thin phase grating.

The optical nonlinearity has sometimes been measured by the same setup as the TG^29^, and here the diffraction light pattern was projected onto a screen to show how the optical nonlinearity was established. The pump pulse was operated at 1 Hz, and the video was recorded. From the video, the snapshots of the brightest patterns were selected. Figure 3(a) shows the diffraction light pattern (left side of the 0^th^ order light), for different pump intensities. For the pump intensity ≤0.10 mJ/cm^2^, only the 1^st^ order and weak intensity of the 2^nd^ order light were recognized, and the pattern suddenly changed for the larger intensities than a threshold around 0.15 mJ/cm^2^. Not only the diffraction intensity increased, but also many diffraction orders were observed.

To examine the nonlinear refractive index, we measured the 1^st^ order diffraction intensity, namely the TG response, as shown in Fig. 3(b). After the response of the initial peak, which corresponds to the molecular dynamics, including disordering and the following molecular rotation and reorientation^18,30^, the TG responses started to increase on a millisecond timescale above the threshold intensity, which was in agreement with the intensity for the diffraction enhancement shown in Fig. 3(a). The peak intensity increased and the peak time was delayed with increasing the pump intensity, and the peak intensity was saturated around 0.55 mJ/cm^2^. The diffraction efficiency of the 1^st^ order diffraction light was obtained by dividing the intensity of the 1^st^ order diffraction by the intensity of the incident light, and this is shown in the right axis in Fig. 3(b). Under the condition of the thin sinusoidal grating, the maximum efficiency is 34% for the phase grating, and the peak intensity for the transient diffraction reached the theoretical limit. The reason why the peak intensity exceeded the theoretical limit is unclear, but there are several possibilities, such that an amplitude grating was included due to the formation of a cis isomer or a different shape of the grating was formed.

**Figure 4:**
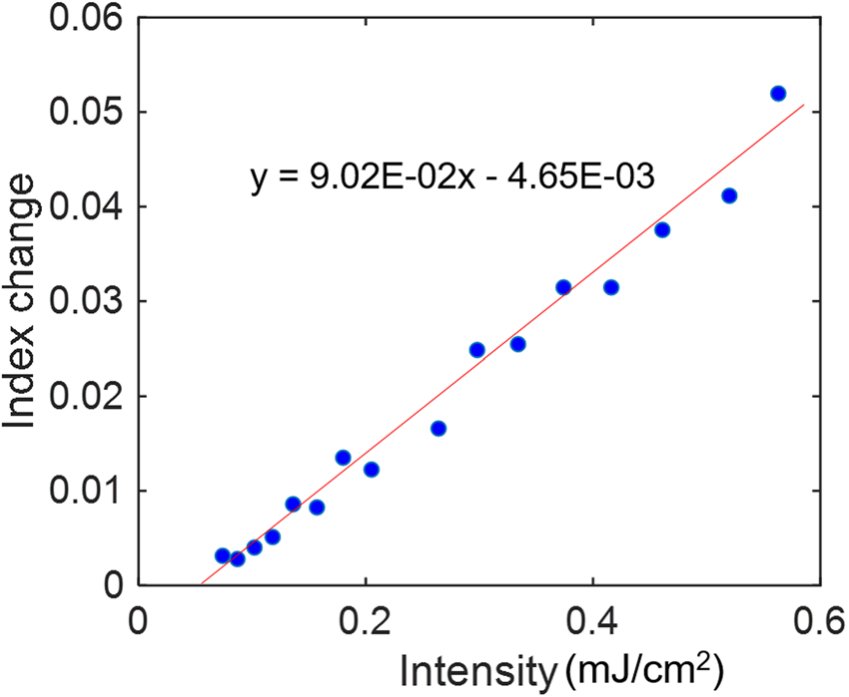
The change in the refractive index change at the peak intensity of the 1st diffraction light of the transient grating response was calculated, and its dependence on the pump intensity is shown. A fitting line for the plot is also shown.

We could obtain the diffraction efficiency by dividing the peak diffraction intensity in Fig. 3 by the incident light intensity measured with the same detector for the TG response. The phase modulation in Eq. (1) can be calculated from the obtained diffraction efficiency, leading to Δ*n* value from Eq. (2). Figure 4 shows the pump intensity dependence of Δ*n*. The refractive index was proportional to the pump intensity above the threshold intensity. By a linear plot using Eq. (3), ${n}_{2}=90(c{m}^{2}/J)$ was obtained. It is noted that the unit of the nonlinear refractive index is different from the general unit, $(c{m}^{2}/W)$, because the diffraction was generated a long time after the irradiation of the pump pulse (pulse width: 4 ns), and *n*_2_ cannot be obtained with the general unit.

**Figure 5:**
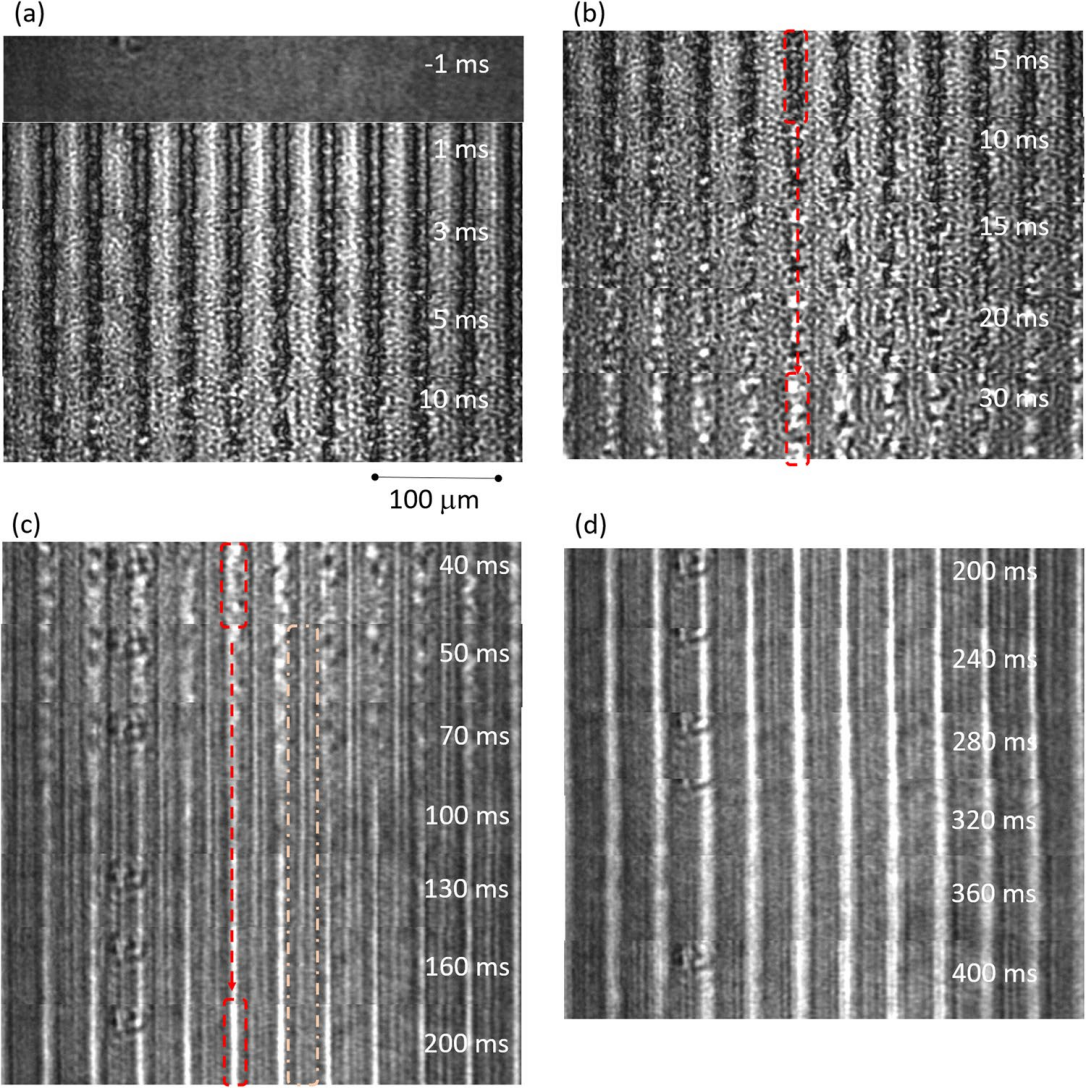
.TG image sequences are shown for the 0.56 mJ/cm^2^ pump intensity for different time regions; (**a**) until 10 ms, (**b**) 30 ms, (**c**) 200 ms, and (**d**) 400 ms. The scale bar corresponds to 100 µm. The prominent change is indicated with a dotted square in (b) and (c). In (b), the dark region corresponding to the isotropic region changed into a disordered structure corresponding to the isotropic droplets. In (c), the disordered structure was changed into a bright line. In between the lines, another wave-fronts can be confirmed from 50–160 ms. The videos are included in Movie 1(c) and Movie 2 in the Supplemental Information.

The movies of the TG imaging for different pump intensities until 100 ms after the pump light irradiation are shown in Movie S1 in Supporting Information (SI). For the pump intensity, 0.56 mJ/cm^2^, the movie of the TG imaging until 500 ms is shown in Movie S2 in SI. The image sequences for the pump intensity, 0.14 mJ/cm^2^, showed a gradual decrease of the stripe contrast by about 50 ms. However, the complicated changes were observed for the higher pump intensity than around 0.2 mJ/cm^2^. Within a few milliseconds, the brightness contrast was formed, but the dark region gradually changed into a bright region within several tens of milliseconds, involving the formation of random domains. Next, the randomly formed domains were merged into bright continuous lines, and the lines became brighter and thinner within several hundred milliseconds, and the contrast gradually decayed by diffusing the line pattern. To clarify the processes, the image sequences for the pump intensity of 0.56 mJ/cm^2^ are shown in Fig. 5.

From the TG image sequences until 10 ms after the pump light irradiation (Fig. 5(a)), the bright/dark stripe pattern was formed. Since the isotropic region became dark in this configuration, the dark regions correspond to the isotropic phase. The formation of the dark regions indicates that the phase transition from nematic to isotropic had already occurred within a millisecond, which agrees with the previous studies showing that the phase transition from the nematic to isotropic finished within hundreds of nanoseconds^20,21^. On the other hand, the bright regions were formed at the non-irradiated regions by the pump light, and the brightness was probably increased because of the temperature rise. Figure 5(b) shows the TG image sequence until 30 ms after the pump irradiation. The dark (isotropic) regions gradually became bright, and it appeared that disordered structure was formed inhomogeneously in the dark region (Compare the dark region in the image at 5 ms with that of 30 ms). The appearance of the disordered strucuture is similar as the appearance observed when the liquid crystal is gradually heated and the phase transition was induced inhomogeneously. Figure 5(c,d) show the TG image sequences until 200 and 400 ms, respectively. The disordered structure was gradually merged into a straight line and became bright, and the brightness reached a maximum around 200 ms, which agreed with the peak maximum for the TG response (t~100 ms) as shown in Fig. 3. (It is noted that the TG response is proportional to ${(\Delta n(t))}^{2}$, and the time constant (τ) of an exponential response, exp(−t/τ), is observed with a response of exp(−t/(τ/2)). There are two observations to be noted; First, the width of the bright line region was thinner than the initially formed isotropic region (Compare the images at 5 ms and 200 ms); and second, the motion of shock-like wave can be noticed in the dotted-line square in Fig. 5(c). Finally, the bright thin line-pattern gradually diffused out over hundreds of milliseconds, as shown in Fig. 5(d).

**Figure 6:**
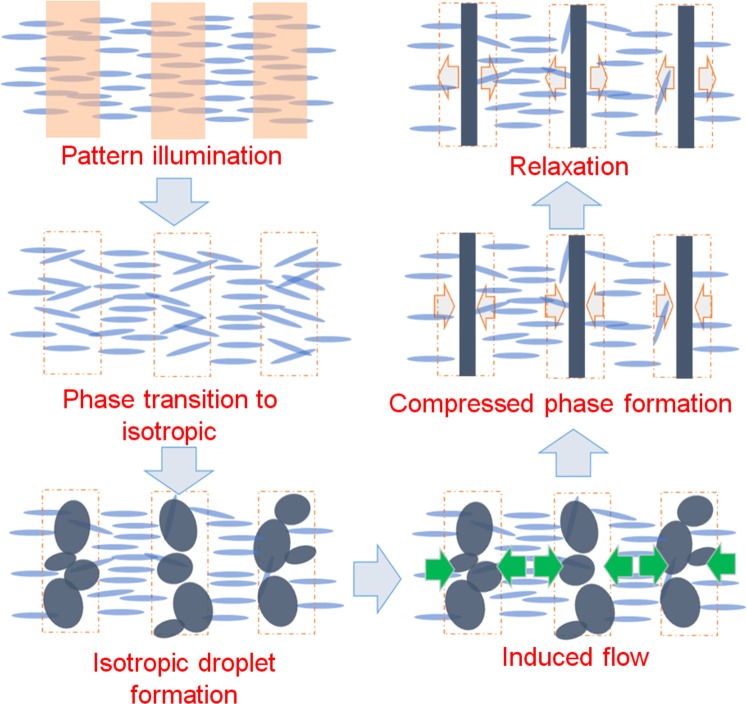
Schematic drawing is shown to describe the formation mechanism of the new phase in the isotropic region generated by the pump pulse light.

The proposed scheme is shown in Fig. 6. On the timescale of milliseconds, the phase transition from the nematic to the isotropic occurred instantly, and the nematic/isotropic stripe pattern is formed. In the isotropic region, the isotropic droplets gradually grew and coagulated to form isotropic indefinite shape until 30 ms. Due to the flow induced by the stress at the interface of the nematic/isotropic region, the indefinite isotropic region became a sharp line until 200 ms, and the phase gradually decayed by diffusion until 500 ms. From the agreement between the emergence time of the sharp line-pattern and the peak time of the diffraction light, we propose that the optical nonlinearity was caused by the line-pattern phase formed in the isotropic region. Lavrentovich, *et al*. reported on the molecular reorientation due to thermal expansion and the subsequent flow^31^. The flow velocity was on the order of 10 μm/s at 20 mm away from the heating position, depending on the temperature gradient and the thermal expansion coefficient. Our observation would have similar points, but our observed process involved the phase transition and the flow was observed around the interface between the nematic/isotropic phase. Since the thermal expansion coefficient diverges for the phase transition temperature^32^, it is difficult to compare the absolute value of the flow velocity, but the mechanism of the flow-induced orientation is supported from this research.

**Figure 7:**
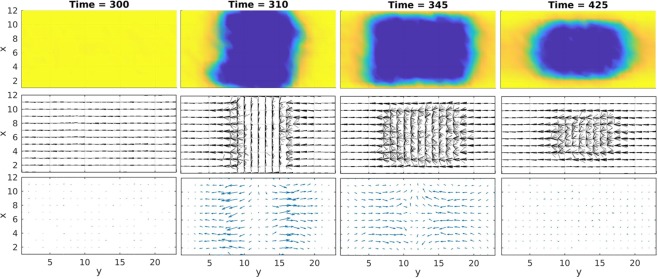
The simulation of the LC order, LC director, and the flow using the Q-tensor model. A part of the LC region (y = 9–15) is photo-excited at Time = 310 instantly. Each row corresponds to the LC order, the LC director, and the flow direction, respectively, and each column corresponds to the snapshots of Time = 300, 310, 345 and 425, respectively.

As an evidence of the flow generation and the subsequent LC alignment by the pulse light irradiation, a theoretical calculation based on the Q-tensor model was performed for a nematic phase close to the phase transition temperature. A part of the LC region (y = 9~15) with a slit shape was suddenly excited, as shown in Fig. 7. The snapshot for the LC order, LC director, and the flow were depicted. The pump excitation on the LC sample is during (Time = 300 to 310). The flow was induced initially outward (at Time = 310) from the light irradiation region, and disordering started around the edge of the isotropic/nematic region. However, at Time = 345, the finite compressibility of the LC fluid suppressed any further expansion, and the flow direction reversed and flew into the isotropic region. This simulation is exactly what we observed in the sequence of the TG imaging, and this calculation supports that our assumption of the flow prediction. However, we could not predict the reorientation of the LC molecules by the flow coming into the isotropic phase, which is expected to show an enhancement of the refractive index change, and the reorientation process must be clarified in future.

**Figure 8:**
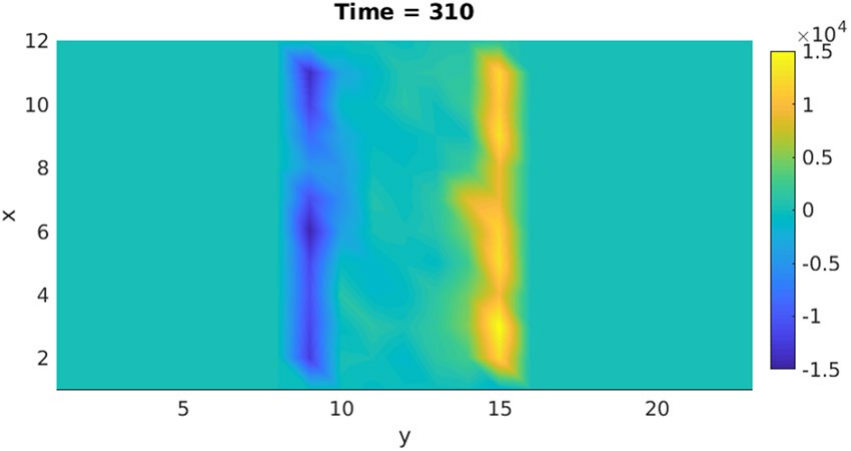
The calculated field-induced stress is shown at Time = 310 during the photo-excitation.

The calculated induced-charge is shown in Fig. 8 when the pump laser is still shined in the LC sample (with +y pump laser field at Time = 310). This charge is temporally generated due to the assembly or alignment of the molecules with charge distribution. The result indicates that the positive and negative charges were accumulated on the right and left side of the boundary of the slit, respectively. Since the Maxwell stress is the product of the charge and the external field, the positive charge flows in the +y-direction, while the negative charge flows in the opposite direction. Thus, they result in strong expansion flows. It is noted that these flow directions are the same for the –y pump laser field because the signs of the accumulated charges are also reversed and as a result, the flow directions do not change.

After this expansion flow, the flow is changed into a compression flow due to the finite compressibility. We propose that this flow caused the reorientation of the LC molecules in the isotropic region. This observation was similar to the earlier work by Khoo *et al*. and Eichler and they explained it as a flow-induced reorientation^10,33^, and Maxwell stress accounts for the generation of LC flows.

It is important to stress that the optical nonlinearity was not induced by a direct interaction between the electromagnetic field and the LC molecules, but rather by the compression induced re-orientation of LCs, causing the optical nonlinearity. Why the LC shows the nonlinear refractive index change remains unclear, but it is possible that the molecular dipole was aligned to make a larger dipole, which is consistent with some reports on the domain formation during the light-induced processes^18,34^. It is possible to induce a larger polarization, causing a large refractive index change by making a larger molecular assembly while maintaining the total charge distribution.

## Conclusion

Using a time-resolved TG phase imaging technique, we studied the mechanism for the emergence of the huge optical nonlinearity of LC after milliseconds of a pump pulse irradiation. The nonlinear polarization was induced in the photo-induced isotropic region, and at the same time, a wavefront propagation (flow) was observed. Based on the theoretical calculation, we proposed that the optical nonlinearity was induced by the reorientation of the LC molecules in the isotropic region induced by the flow generated at the interface of the disordered/ordered region.

## Experiment

The pump light was Nd:YAG pulse laser (pulse width: 4 ns, wavelength: 355 nm) (GAIA, Rayture Systems) and the probe light was an Nd:YVO_4_ laser (wavelength, 532 nm; irradiation intensity, 5 mW) (JUNO, SOC). The diameter of the irradiated area by the pump pulse was 5 mm. The interval of the pump pulse was long enough that the baseline went back to the original value, which was typically 1 second. The irradiation intensity was changed in the range of 0.05–1 mJ/pulse with the 75-μm grating spacing. Due to the reduction optics, the actual grating spacing was 36 μm. A CMOS camera (MV1-D1024E-160, Photon Focus) was used for the time-resolved imaging. After a trigger signal of the pump pulse, a sequence of images was stored by a computer. The time resolution was limited by the exposure time of the camera, 11 μs.

MBBA (Tokyo Kasei) was used as purchased. The sample was put into an LC cell (E.H.C) with a sample thickness of ~3 μm, with a rubbed polyimide film for a planer alignment inside, and the corresponding absorbance was 0.28 for the pump light. The LC cell was covered by an aluminum frame, whose temperature was controlled by a heater controller (TC200, Thorlabs). The temperature was set at 30.0 °C, where the nematic-isotropic phase-transition temperature (T_NI_) was 44.0 °C, measured by a differential scanning calorimetry.

## Supplementary information

Supporting information

Movie S1(a)

Movie S1(b)

Movier S1(c)

Movie S2
