# Supplementary material for: Origin of optical nonlinearity of photo-responsive liquid crystals revealed by transient grating imaging
Source: Sci Rep. 2019 Apr 8;9:5754. doi: 10.1038/s41598-019-42140-x (PMC6453968; doi:10.1038/s41598-019-42140-x)
Supplement: Supplementary file 1 — Supporting information [file 41598_2019_42140_MOESM1_ESM.pdf]

Supplementary Information of
Origin of optical nonlinearity of photo-responsive liquid crystals revealed by transient grating imaging

Kenji Katayama, Daiki Kato, Kin-ichiro Nagasaka, Minako Miyagawa and Woon Yong Sohn, Kuang-Wu Lee

Video information

Movie.S1 The videos of the TG imaging for different pump intensities are shown for the pump intensity (a) 0.14, (b) 0.37, (c) 0.56 mJ/cm^2 and until 100 milliseconds. The grating spacing on the sample was 36 μm.

Movie.S2 The video of the TG imaging for the pump intensitiy, 0.56 mJ/cm^2 and until 500 milliseconds is shown. The grating spacing on the sample was 36 μm.
